# Trajectory Design for UAV-Based Data Collection Using Clustering Model in Smart Farming

**DOI:** 10.3390/s22010037

**Published:** 2021-12-22

**Authors:** Tariq Qayyum, Zouheir Trabelsi, Asad Malik, Kadhim Hayawi

**Affiliations:** 1 Department of Computing, School of Electrical Engineering and Computer Science, National University of Sciences and Technology (NUST), Islamabad 44000, Pakistan; tqayyum.msit16seecs@seecs.edu.pk (T.Q.); asad.malik@seecs.edu.pk (A.M.); 2 College of Information Technology, United Arab Emirates University, Abu Dhabi P.O. Box 17551, United Arab Emirates; 3 College of Technological Innovation, Zayed University, Abu Dhabi P.O. Box 144534, United Arab Emirates; abdul.Hayawi@zu.ac.ae

**Keywords:** clustering, fog computing, smart farming, swarm UAVs, IoT, sensors

## Abstract

Unmanned aerial vehicles (UAVs) play an important role in facilitating data collection in remote areas due to their remote mobility. The collected data require processing close to the end-user to support delay-sensitive applications. In this paper, we proposed a data collection scheme and scheduling framework for smart farms. We categorized the proposed model into two phases: data collection and data scheduling. In the data collection phase, the IoT sensors are deployed randomly to form a cluster based on their RSSI. The UAV calculates an optimum trajectory in order to gather data from all clusters. The UAV offloads the data to the nearest base station. In the second phase, the BS finds the optimally available fog node based on efficiency, response rate, and availability to send workload for processing. The proposed framework is implemented in OMNeT++ and compared with existing work in terms of energy and network delay.

## 1. Introduction

The rapid advancement in wireless sensor networks (WSN) has emerged with an assortment of curiosity [1,2]. The inclusion of unmanned aerial vehicles improves the coverage area as well as communication between devices [3]. The most common applications of UAV in data collection are covered in [4,5]. The clustering schemes on WSN are used for data collection to preserve energy and reduce communication overhead [6]. In most cases, sensors are deployed at difficult-to-reach locations. Sometimes, these locations include hilly areas and large farms where nature hurdles such as trees, small mountains, or unexpected slopes cause signal distortion. In such cases, it is difficult to gather data. However, the use of UAVs in such areas can reduce the cost and increase the efficiency of data collection [7]. Data collection using UAV in large-scale smart farms faces considerable challenges in terms of lack of connectivity, interoperability, data storage, limited energy resources, latency, and data processing power. Furthermore, data processing in delay-sensitive applications is another big challenge. In conventional data collection schemes, the UAV collects data and returns it back to the base station to offload collected data for processing. This process is repeated until all the farms are traversed. This back and forth traveling adds additional delay and consumes more energy [8]. Moreover, smart farm applications also require quick responses to generate triggers for the farmers. Moreover, the data collection by visiting every sensor in the field adds additional energy cost; thus, clustering techniques are adopted for the sensors nodes [9]. UAVs move sensor to sensor, cluster by cluster to collect data by communicating with sensors directly or acquiring the data from cluster heads (CHs). Data collection directly from sensors is an easy task, but it consumes more energy, time, and travel distance for the UAV as compared to the later technique where data are relayed through the CH. However, the second technique is also not very efficient because it yields the problem of a single point of failure, network congestion, and bottleneck at the CH. This problem can be resolved by adopting a hybrid technique where CH helps in creating a reliable connection between sensors and UAV, and the UAV receives data from sensors at a single optimal position [10]. The proposed technique is based on a hybrid mechanism where CH is selected based on centrality. With this technique, the UAV can collect data from the entire cluster by hovering at a single position, i.e., at the top of the CH.

Fog computing is an emerging technology that brings intelligence closer to the data source. Usually, the IoT devices have very limited computation resources, and to compensate, fixed nodes are placed at the network edge to perform complex computations instead of sending the raw data to cloud data centers. Hence, only a small amount of the information is forwarded to the cloud via the Internet, which also resolves the bandwidth issues and network latency [11]. In a typical fog computing architecture, the base stations (BS) can act as a broker node, deployed at the edge of the network, and multiple fog servers are connected with it. When a task is received at BS, it schedules that task to a nearby fog node. Such a resource sharing paradigm is categorized into resource allocation, provisioning, resource sharing, and workload allocation [12]. The main objective is to share available resources efficiently and provide service in a latency-sensitive environment to the end device. In conventional data collection frameworks [9,10,13,14], the data are collected and processed in batches, which creates extra delay; however, here we introduce a framework for data collection, processing in a parallel manner. The UAV collects data from clusters, shares it to nearby BS for processing, and continues collecting new data. This parallel working reduces the extra delay that improves the efficiency of latency-sensitive applications for smart farming.

**Contributions**—The proposed framework handles the problem of timely data collection from randomly deployed sensors and data processing for delay-sensitive applications for smart farming. Here we utilized the concept of fog computing. In the proposed work, the UAV submits data to a nearby base station for processing and continues collecting data from other sensors. This model processes and collects data simultaneously, which reduces the unnecessary delays that exist in conventional data collection frameworks. Further, we proposed an energy-sensitive clustering algorithm for sensors deployed in a random smart farming environment that selects cluster heads with minimal iteration. Finally, we improved data processing by proposing a multi-objective scheduling algorithm among available fog servers. The features of the proposed solution are listed as:

1. We proposed an energy-sensitive RSSI-based clustering algorithm for smart farms that selects CHs based on centrality.
2. Using the CHs’ positions, we have adopted the modified Dijkstra and genetic algorithm (GA) to find the optimal trajectory for data collection.
3. Further, we proposed a data scheduling algorithm based on a multi-objective trade-off between the network cost and availability of the fog servers. The UAVs offload the data to the nearby base station (BS). The BS distributes this data to the fog nodes available near to the end-users.

**Paper Organization**—The rest of the paper is organized such that Section 2 discusses the state-of-the-art literature review about current work on data collection and scheduling using UAVs and fog servers. The system model is discussed in Section 3. The system components are discussed in Section 4. Finally, the accuracy and performance of the proposed system are discussed in Section 5, followed by the conclusion in Section 6.

## 2. Related Work

In this section, recent contributions in clustering-based networks, remote data collection, and data scheduling for streaming data from IoT devices are covered.

**Clustering**—In WSN, many sensors are deployed; in such scenarios, clustering is used to preserve energy and perform certain tasks. Maheswari et al. [15] proposed a routing protocol based on fuzzy logic. The proposed scheme is a two-level hierarchical approach where appropriate parameters are used to select cluster head and supercluster head. The performance of the proposed scheme is evaluated in terms of node death using actual hardware devices and through simulation. Leo et al. [16] proposed a clustering algorithm referred to as Regional Energy-Aware Clustering with Isolated Nodes (REAC-IN). In the proposed algorithm, the cluster head is selected based on the weight that depends on the regional average energy and residual energy of the nodes. In simulation-based evaluation, the proposed algorithm outperformed other traditional algorithms. Li et al. [17] proposed a clustering scheme where they classified nodes into three types: regular, suspicious, and malicious nodes. The scheme isolates the malicious nodes from the networking to avoid selective forwarding attacks and later screens out malicious nodes. The punishment and reward mechanism is adopted to minimize the trust values and expected revenues of the attackers. Cho et al. [18] proposed a calibration technique for WSN. The clustering strategy is used to do robust estimation efficiently. They proposed multiple algorithms to calculate the lower bound value of the redundancy degree, whereas another algorithm is proposed to estimate the least-trimmed squares (LTS). Wang et al. [19] proposed a particle swarm optimization (PSO)-based clustering algorithm for WSNs using mobile sink node. The proposed algorithm is evaluated with extensive simulation, and performance is compared with other routing algorithms. Omeke et al. [20] proposed a clustering protocol for underwater wireless sensor networks. The k-means clustering algorithm is enhanced and the cluster head is selected based on the weight. The weight is based on the residual energy value of the sensor and the position of a node in the cluster. To make the cluster scaleable, the elbow method is used for dynamic selection of the optimal number of clusters. The proposed solution outperformed when compared with the low-energy adaptive clustering hierarchy (LEACH) and optimized version of LEACH. Ni et al. [21] proposed a fuzzy clustering-based algorithm to form the initial clusters based on the geographical positions, and the probability that a node belongs to a particular cluster is calculated. Moreover, a fitness function is formed based on the distance factor and energy consumption of WSN. Finally, the cluster is determined by an improved particle swarm optimization algorithm.

**Data Collection**—In a large-scale WSN network where sensors are deployed randomly without following a topology, techniques that are based on the UAV can be used to collect data in an energy-sensitive and efficient way [22]. The existing data collection techniques focus on power controlling [13], energy consumption reduction [23], completion time minimization [14], and trajectory distance minimization [24]. Samir et al. [5] proposed a trajectory planning framework to collect data using UAVs from IoT devices. A branch, reduce, and bound algorithm is used to solve the subproblems, and then they developed a sub-optimal algorithm for a larger network. Liu et al. [10] studied an age-optimal trajectory problem and designed two trajectories for the maximum age of information and the average age of information. These trajectories are used to minimize the oldest information and average age of information of all sensors. Further, generic algorithms and dynamic programming algorithms are used to estimate the trajectories. Pang et al. [9] proposed a cluster-based data collection for rechargeable sensors. They used multiple UAVs to collect data by visiting the clusters and then recharging cluster nodes. A greedy algorithm and one-side matching algorithm is developed to solve the problem in a distributed manner. Cheng et al. [25] proposed a UAV trajectory optimization technique to collect data from multiple sensors using UAVs. They also optimized the mixed-integer nonconvex problem by transforming it into two convex problems. Ebrahimi et al. [26] proposed a reinforcement learning-based approach for object localization using UAV. Instead of GPS, the RSSI of the received signal is used to locate objects on the ground. Similarly, Spyridis et al. [27] proposed deep-learning-based clustering scheme to trace mobile sensor nodes using UAV. Ma et al. [28] studied a data collection framework using UAV in an architecture-less environment where mobility changes over time. They proposed four algorithms for data collection considering contract duration time between UAV and sensors, and data rates. The results are compared in a simulation manner. Yang et al. [29] proposed a machine-learning-based prediction method for path loss and delay spread in air-to-ground millimetre-wave channels. Zhu et al. [30] proposed a new 3D non-stationary, geometry-based stochastic channel model for UAV-ground communication systems. Both [29,30] worked on channel loss and delay spread of wireless channels, whereas we used low altitude UAVs with a refined trajectory to ensure LOS communication between UAV and sensors.

Zhan et al. [31] proposed an energy minimization technique using successive convex and alternating optimization for a UAV-based data collection system in an IoT environment. The simulation results proved the performance when compared to other schemes. Gong et al. [32] proposed a flight time minimization scheme for UAV-based data collection system in WSN. The whole trajectory is divided into multiple intervals. The transmit power of the sensors, speed of UAVs, and data collection intervals are optimized. It was concluded that the speed of the UAV is proportional to the distance between sensors and the energy of the sensors. The above-mentioned solutions collect data by visiting the sensor nodes physically and collecting via CH. This causes multiple issues such as a single point of failure, an unnecessary bottleneck, and energy crises at the CH.

**Data Scheduling**—Mostly, research on fog computing has focused on the offloading and scheduling strategies to help resource-limited IoT devices. Liu et al. [33] proposed a low latency workload offloading in mobile edge computing to yield low latency communication. The proposed framework focuses on the trade-off between energy and latency. The task is divided into multiple sub-tasks and offloaded to multiple fog devices. Abedi et al. [34] proposed a resource offloading strategy in fog-cloud-based networks using artificial intelligence. The proposed strategy focuses on distributing the tasks to fog servers and cloud servers to reduce the end-to-end delay. Gu et al. [35] proposed a resource management framework for fog-based medical cyber-physical systems. The main contribution is in terms of virtual machine placement, task distribution, and BS association. Shih et al. [36] studied the fog-based solution for low-latency applications. A framework is discussed to trade-off among many performance parameters such as computing cost, performance, and communication cost. Deng et al. [37] proposed a workload allocation framework for fog-cloud-based architecture to balance between power consumption and delay. A trade-off is investigated between power consumption and network delay. The problem is formulated and then divided into three sub-problems. Finally, the solution is bench-marked using simulation in terms of latency, bandwidth, and power consumption. Brogi et al. [38] instigated the application placement strategies using their proposed framework ForgTorchII. FogTorchII is capable of finding the multiple deployment strategies for fog network environments, which attain QoS, hardware, and software requirements. Sun et al. [39] proposed a crowdfunding algorithm for fog computing to utilize the unused resources. An incentive mechanism is adopted in the algorithm to encourage resource owners to volunteer more resources, and the simulation results proved that the proposed algorithm was effective in terms of reduction in SLA violation rate and enhancing the task completion rate. Yang et al. [40] proposed an energy-efficient task scheduling algorithm for homogeneous fog networks called MEETS. The proposed algorithm investigates the optimal resource-sharing solutions for nearby homogeneous fog nodes. The simulation results are compared with traditional task scheduling algorithms and concluded that the proposed algorithm outperformed in terms of energy efficiency and scheduling performance. The best case in fog computing offloading is when the workload is scheduled/offloaded to a single node as discussed in  [33,34,35], whereas some researchers focus on distributing the task into multiple sub-tasks and offloading them to multiple fog nodes in a horizontal manner as discussed in  [36,37,38]. However, another great solution is hybrid/parallel placement, where both fog nodes and cloud servers are used to process the tasks [39,40].

The above-mentioned task scheduling solutions ignore the important parameters such as the availability of the fog nodes and network cost. The fog nodes sometimes go offline due to many factors which include power limitations, connection loss, and workload. Furthermore, in a realistic fog network, the communication cost is different for all fog nodes due to distance, data rate, error rate, and bandwidth, which is often ignored while considering offloading/scheduling tasks at nearby fog nodes.

## 3. System Model

**Figure 1:**
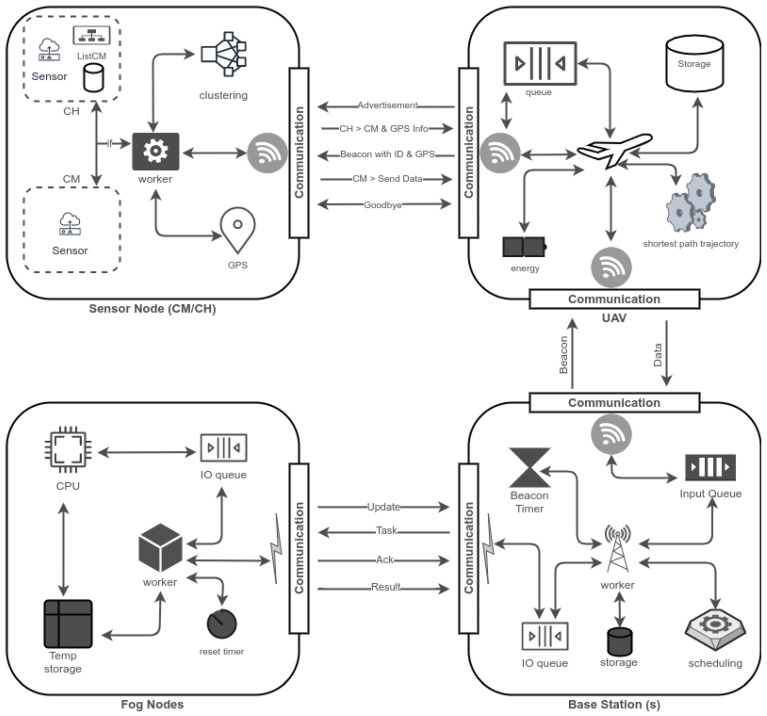
Working architecture—The working of different components of the system with sub-components. The sensors send data to UAV and UAV offload received data to a nearby base station that further schedules that to its fog members for further processing.

This section covers the system model used for the proposed framework. The abstract-level working architecture of the proposed system is given in Figure 1. The summary of notations used in this paper is given in Table 1.

**Deployment Model**—The sensors are deployed randomly across the farming area. The set S represent the sensor nodes S={1,2,3,…,|N|} along with coordinates in three-dimensional space as $X=\{s_{1},s_{2},s_{3},\ldots ,s_{\left|N\right|}\}$, where $s_{\left|N\right|}\in R^{3\times 1}$. The data collection is performed from the randomly deployed sensors in the remote areas, and no physical network is available. The data are collected from the sensors at typical 2.4 GHz of the cellular network.

**UAV Mobility Model (UMM)**—The UAVs are initializing the CH discovery mechanism at the starting point.

(1) $$\begin{array}{c} U_{s}=\left[\begin{array}{c} {SF}_{0}\\ {SF}_{0}\\ \Upsilon_{U} \end{array}\right] \end{array}$$

where the initial position of the UAV is represented as $U_{s}$, and $\Upsilon_{U}$ represents the initial height of the UAV. So, the starting coordinates are (0,0,$\Upsilon_{U}$). After the discovery phase, the final position is denoted as:

(2) $$\begin{array}{c} U_{f}=\left[\begin{array}{c} {SF}_{x}\\ {SF}_{y}\\ \Upsilon_{U} \end{array}\right], \end{array}$$

Here $U_{f}$ is the final position. After reaching the x-axes boundary of the form, the UAV shifts $Q_{Y}$ depending on the y-axis. The $Q_{Y}$ is calculated as:

(3) $$\begin{array}{c} Q_{Y}=\left\{\begin{array}{cc} Q_{Y_{S}}*2, & Q_{Y_{S}}\leq Q_{Y_{U}}\\ Q_{Y_{U}}*2, & Q_{Y_{Y}}<Q_{Y_{S}} \end{array}\right., \end{array}$$

where $Q_{Y_{U}}$ and $Q_{Y_{S}}$ are the displacement of the y-axis depending on UAV’s and sensors’ transmission range, respectively. This displacement value is calculated as:

(4) $$Q_{Y_{S}}=\sqrt[{2}]{\varrho_{Sr}^{2}-\Upsilon_{U}^{2}},$$

(5) $$Q_{Y_{U}}=\sqrt[{2}]{\varrho_{Ur}^{2}-\Upsilon_{U}^{2}},$$

where $\varrho_{Sr}$ and $\varrho_{Ur}$ are the transmission radius of sensors and UAVs, respectively. The UAV broadcasts a *hello* message after an interval, and this interval is calculated such that all deployed sensors are in the transmission radius of the UAV. This interval is calculated as:

(6) $$br_{p}<br_{n}<\left({br}_{prev}+\left(\frac{U_{R}^{Q_{y}/2}}{\upsilon_{U}}\right)\right),$$

where $br_{p}$ and $br_{n}$ are the time of the previous and next hello message, whereas $\upsilon_{U}$ is the speed of UAV and $U_{R}^{Q_{y}/2}$ is the range of transmission. The ratio $\left(\frac{U_{R}^{Q_{y}/2}}{\upsilon_{U}}\right)$ is the duration when UAV and sensors are in range to effective transmission. This condition in Equation (6) must meet to ensure the beacon delivery to all sensors.

**Data Collection Model**—A sensor broadcasts a hello message beacon along with its ID and geo-location. The receiving sensor nodes form a list of its neighboring nodes $L_{ngr}^{S}$ based on RSSI values. Both the UAV and deployed sensors are capable of calculating the RSSI, as it is trivial for a WSN device to calculate the RSSI, which is a legitimate assumption [41]. Moreover, the proposed system assumes a low-altitude UAV, the sensors are clustered together and UAV fine-tunes the position for data collection. With this ability, despite some natural obstacles, the UAV can establish LOS communication. Thus, a sensor node developed a list $G_{ngr}^{S}$ of its all neighbors along with their geo-locations. The node broadcasts its cost value to all neighbors. A polygon structure of the sensor nodes is formed, where the area of the polygon area is calculated using the typical formula:

(7) $$Area=\frac{1}{2}\sum_{j=0}^{K-1}\left(G_{j}^{X}\cdot G_{j+1}^{Y}-G_{j+1}^{X}\cdot G_{j}^{Y}\right)$$

where $G^{X}$ and $G^{Y}$ are the lists of geo-positions of nodes located at the polygon’s edge sorted by x-axis and y-axis, respectively. The *K* represents the number of sensor nodes taking part in polygon formation. The centroid $\Theta_{x,y}$ is calculated as:

(8) $$\Theta_{x}=\frac{1}{6Area}\sum_{J=0}^{K-1}\left(G_{J}^{X}+G_{J+1}^{X}\right)\left(G_{j}^{X}\cdot G_{j+1}^{Y}-G_{j+1}^{X}\cdot G_{j}^{Y}\right),$$

and

(9) $$\Theta_{y}=\frac{1}{6Area}\sum_{J=0}^{K-1}\left(G_{J}^{Y}+G_{J+1}^{Y}\right)\left(G_{j}^{X}\cdot G_{j+1}^{Y}-G_{j+1}^{X}\cdot G_{j}^{Y}\right),$$

and by combining the x-axis and y-axis:

(10) $$\Theta_{x,y}=\left(\Theta_{x},\Theta_{y}\right)$$

For every node, a probability is computed to become a CH, which is calculated as:

(11) $$P_{CH}=\max\left(\left(1-\frac{\chi_{a,b}}{\varrho_{Sr}}\times \zeta \right),\kappa_{min}\right)$$

where the transmission radius of a sensor is represented by $\varrho_{Sr}$. The number of iterations in clustering depends on the normalization factor ζ. The $\kappa_{min}$ is assigned as the CH when the central bias is lower than a provided threshold. The distance between central node *c* and current node *n* is calculated by Euclidean distance and represented by $\chi_{c,n}$, which can be calculated as:

(12) $$\chi_{c,n}=\sqrt{{\left(c_{x}-n_{x}\right)}^{2}+{\left(c_{y}-n_{y}\right)}^{2}}.$$

For data collection, the shortest patch trajectory is calculated, and according to [42], the fitness function of the UAV trajectory is given as

(13) $$d\left(O\right)=\varepsilon_{\left(S_{e},O_{1}\right)}+\varepsilon_{\left(O_{\left|O\right|},S_{e}\right)}+\sum_{i=1}^{\left|O\right|}\varepsilon_{O_{i},O_{i+1}}$$

where $d\left(O\right)$ represents the total distance covered by the UAV in a single round. $\varepsilon (S_{e},O_{1})$ represents the distance between starting data collection position to the first CH’s position $O_{1}$. $\varepsilon_{\left(O_{\left|O\right|},S_{e}\right)}$ represents the distance between last data CH’s position and final point of the farm.

**Delay Model**—The delay model is based on delay occur during data collection and due to the data execution at the fog, which is explained below:

*Data Collection*—

(14) $$\varphi_{\forall }=\sum_{r=1}^{n}\left(\frac{g\left(G_{CH}\right)}{\upsilon_{U}}+\sum_{i\in C}\sum_{k\in i}D_{k}^{i}\right),$$

where $\varphi_{\forall }$ is the time required for a UAV to collect data from all the sensors for *n* number of rounds. $G_{CH}$ is the list of CHs’ positions, $\upsilon_{U}$ is the speed of UAV, *C* is the number of clusters, *c* is the current cluster with *k* number of sensors, and $D_{k}^{c}$ is the transmission delay between kth sensor of cluster *c* and UAV.

*Data Execution*—The execution delay of the system calculated as [43]:

(15) $$\begin{array}{c} D_{\forall }=\sum_{r=1}^{n}\sum_{b=1}^{B}\left(\sum_{\omega \in \Omega }D\left(p\right)+\sum_{\omega \in \Omega }D\left(q\right)+\sum_{\omega \in \Omega }D\left(t\right)\right), \end{array}$$

where D(p), D(q), D(t), and D(pr) are the processing, queuing, transmission, and propagation delays of workloads ω in list Ω, respectively. These delays are calculated as:

(16) $$D_{p}\left(\omega \right)=\frac{A_{\omega }}{\delta_{v_{f}}},$$

where $A_{w}$ is traffic load and $v_{f}$ is the processing rate of a fog node.

(17) $$D_{q}\left(\omega \right)=\frac{\lambda_{f}S_{\omega }}{\delta (e)},$$

where λ(f) is the arrival rate at a fog node, $S_{\omega }$ is the processing required by a workload ω, and δ(e) is the transmission rate of the link. Finally, the transmission delay is calculated as

(18) $$D_{t}\left(\omega \right)=\frac{A_{\omega }}{\delta (e)},$$

and the total application delay is calculated as

(19) $$D_{A}\left(\omega \right)=Max\left(D_{\forall },\varphi_{\forall }\right),$$

**Data Scheduling Model**—We consider that a UAV offloads a list of workloads $\Omega =\{\omega_{1},\omega_{2},\omega_{3},\ldots ,\omega_{n}\}$. Each BS is equipped with a number of fog devices $ϝ=\{f_{1},f_{2},f_{3},\ldots ,f_{m}\}$. These fog nodes have different power efficiency ${ℸ}_{i}$, response rate $\psi_{i}$, and availability $A_{i}$.

*Power Efficiency*—The power efficiency is the amount of energy consumed during processing a single unit of received workload [44]. This efficiency is a combination of power usage effectiveness ${℧}_{i}^{e}$, static ${℧}_{i}^{s}$, and dynamic power usage ${℧}_{i}^{d}$ of the ith fog node. The static power usage is sometimes referred to as power leakage produced by the leakage currents, whereas the dynamic power consumption is caused by the circuit currents, calculated by the activity of the fog nodes.

The total power consumption ${℧}_{i}^{T}$ of a fog node per unit time is calculated as:

(20) $${℧}_{i}^{T}={℧}_{i}^{e}\left({℧}_{i}^{s}+{℧}_{i}^{d}\lambda_{j}\right),$$

where $\lambda_{j}$ is the average arrival rate at fog node $f_{j}$.

The power efficiency of a fog node is determined by

(21) $$\Psi_{i}=\frac{{℧}_{i}^{T}}{\lambda_{i}}={℧}_{i}^{e}\left(\frac{{℧}_{i}^{s}}{\lambda_{i}}+{℧}_{i}^{d}\right).$$

*Response Rate*—Let $\hbar_{i}$ be the round trip time (RTT) between a fog node $f_{i}$ and BS. The transmission delay $\tau_{i}$ between an ith fog node and BS can be calculated by the fraction of workload size and bandwidth. Hence, the response time of a fog node can be calculated as:

$$\psi_{i}=\hbar_{i}+\left(\frac{1}{\tilde{\lambda_{i}}-\lambda_{i}}\right)+\tau^{f}.$$

where $\tilde{\lambda_{i}}$ is the maximum amount of workload that can be computed on a fog node $f_{i}$.

*Availability*—The availability of a fog node can be calculated by dividing uptime by the total sum of uptime and downtime [45]. The fog nodes periodically send beacons to the BS, and based on those beacons, the BS calculates the availability of each fog node and updates it in the fog table.

## 4. Proposed Framework

The proposed framework comprises several main entities which are discussed below.

**Farming Area**—The farm is the main component of the proposed work. Other devices are used to gather data from farms for the knowledge-aware decision process. In this work, we have assumed that the farming area can be a flat surface or comprised of hilly areas. However, in a hilly scenario, there are maybe natural obstacles such as trees, and mountains are present. These obstacles cause reflection, diffraction, and scattering. The only communication affected by these obstacles is ground-to-ground communication that sensors perform with each other [46]. Therefore, the communication between air-to-air or air-to-ground is not affected by these obstacles.

**Sensors**—Sensors are the actual data generators; the entire system is based on the data generated through sensors and IoT devices. In this work, static sensors are modeled and deployed all over the farming areas. Further, it is assumed that the nodes are aware of their coordinates with the help of a GPS sensor [47]. The sensors deployed in the farm are heterogeneous in nature, which means they have different computation power, storage, and energy. These sensors are deployed randomly across the application area. The random placement helps in creating a realistic smart farming environment. Further, the clustering algorithm makes a group of these sensor nodes and also selects the centrality. The sensor nodes are classified into two categories: cluster members and cluster head (CH). Each cluster of the sensors has one CH that is in the range of all cluster members. Here, we assumed sensor nodes are non-reachable. It is also assumed that sensors are capable of controlling the transmission power of the transmitter to save energy.

**Unmanned aerial vehicles (UAVs)**—UAVs fly across the fields to collect data, which are further transmitted to fog nodes for processing. Here, we assumed that UAV flying paths are defined in such a way that they can complete one round of data collection without energy issues. The UAVs are recharged after one round [48]. In existing work, researchers use solar power and battery replacement strategy after each round [49], which can be one of the future directions in this work. Further, we used a quad-copter UAV that can stay in a stationary position for a specified period and can turn with the least angle [50].

**Base Stations (BS)**—Base stations are the intermediate devices between UAVs and fog nodes and are also referred to as fog brokers. The UAVs offload data to base stations using a wireless channel. The data are further transmitted to fog nodes for faster computations. The following constraints apply to base stations. These base stations are deployed across the network area, which consists of the static nodes. The base station is equipped with AC power and it is also assumed that a backup energy source is available in case of power failure. The power and backup management are out of the scope of this research, but many researchers already solved this problem [51]. The base stations are heterogeneous in nature and have different storage and computation power. Each base station is linked with a different number of fog nodes. Additionally, base stations are capable of buffering the incoming data. The BS works in a real-time environment and reschedules the incoming data to fog nodes in a real-time manner.

**Fog Server**—Similar constraints are applied to fog nodes as of BS. All fog nodes are static in nature, equipped with a power source. They are heterogeneous in nature, and the computation and storage resources can vary. Moreover, each fog node has its availability, resources, and cost.

### 4.1. Design and Implementation

The system architecture is explained in Figure 1, where the main components of the system—sensors, UAV, BS, and fog nodes—communicate with each other to form a complete system. A wireless channel is used to enable communication between sensors and UAV and between UAV and BS, whereas a wired communication medium is used to link BS and fog servers. Sensors are equipped with GPRS sensors, and they form clusters based on their geo-location and RSSI values. The UAVs hover above the sensors to collect data and offload that data to nearby BS. The BS converts the received data into workloads in terms of millions of instructions per second (MIPS) and schedules them to the fog servers. The proposed system is classified into two main phases: data collection and data scheduling. The data collection phase is sub-categorized into initialization, service discovery, and collection. In the initialization section, the clusters are formed through a clustering algorithm and cluster heads (CHs) are identified. The CHs are selected based on node centrality. Thus, it helps UAVs to collect data through CMs by hovering over the CH. This way, the CH’s role is minimized and the central point of failure is eliminated. Unlike conventional data collection techniques, the CH only helps in creating an optimal area where the UAV can access all CMs from a single position. In conventional data collection frameworks, data are collected via CH [49,52]. Collecting data using CH is not feasible for large-scale networks where a large number of sensors are deployed because it will increase the data collection time and increase the distance traveled by UAV. Moreover, it will increase the energy consumption and will not be economical in terms of energy [53]. Further, to determine the CHs position, the UAVs broadcast *hello* message where only CH responds to it. The location of the CHs is calculated using an S-path model discussed in [54]. The technique keeps tracking the distance of all CHs. The UAV starts collecting the data by visiting the location of a CH.

In the data collection phase, the UAVs fly based on the shortest path which is defined using the modified Dijkstra algorithm [55]. However, in data scheduling phase, there is a set of base stations $B=\{b_{1},b_{2},b_{3},\ldots ,|m|\}$, where each base station manages a set of fog nodes $F=\{f_{1},f_{2},f_{3},\ldots ,|n|\}$. The BS buffers the incoming data into its queue and schedules it to the fog node with the help of a scheduling algorithm as stated in Algorithm 1.

**Data Collection**—This section explains in detail the data collection procedure using the UAVs. The data collection is performed via a modified Dijkstra algorithm. The Algorithm 4 gives the shortest path trajectory among discovered CHs. Once a UAV visits a CH’s position, it sends a ping message to CH. The CH generates the advertisement message to all CMs to inform the address of the UAV for data transmission. The CMs send the data directly to the UAV.
**Algorithm 1** Workload Scheduling Algorithm**Input**$ϝ=\{f_{1},f_{2},f_{3},\ldots ,f_{m}\}$                                                                                                       ▹ List of fog nodes$\Omega =\{\omega_{1},\omega_{2},\omega_{3},\ldots ,\omega_{n}\}$κ                              ▹ A trade-off value of efficiency                      ▹ List of workloads**Output** Execute the workload1:$S,S_{A},S_{E},S_{R}\leftarrow \emptyset$2: **for each** ω∈Ω **do**3:    $Demand_{A}\leftarrow CalAvg\left(F.A\right)$4:    $Demand_{E}\leftarrow CalAvg\left(F.E\right)$5:    $Demand_{R}\leftarrow CalAvg\left(F.R\right)$                                                                                                 ▹ Calculate average6:    **for each** f∈ϝ **do**7:         **if** $f_{availability\geq Demand.A}$ **then**8:                           $S_{A}\leftarrow f$9:         **end if**10:        **if** $f_{responseTime\leq Demand.R}$ **then**11:                           $S_{R}\leftarrow f$12:        **end if**13:        **if** $f_{efficiency\geq Demand.E}$ **then**14:                           $S_{E}\leftarrow f$15:        **end if**16:    **end for**17:    $S\leftarrow S_{A}\cap S_{E}\cap S_{R}$                                                                        ▹ Get intersection of fog nodes in three sets18:    **if** S==∅ **then**19:        $demand_{E}\leftarrow demand_{E}-\kappa .demand_{E}$                                     ▹ reduce the efficiency value by factor κ20:        go to step 13;21:    **else**22:        $Send(\omega ,Min\sum_{J=0}^{n-1}\left(S_{A}^{j},S_{E}^{j},S_{R}^{j})\right)$                                                          ▹ Send workload to the best node23:    **end if**24:**end for**

**Clustering**—The clustering of deployed sensors is performed in a distributed manner. An energy-aware clustering algorithm [56] is adopted for clustering. The algorithm forms the clusters and selects CH such that it is located at the central position of the cluster. The CH can communicate with all CM; hence, if the UAV arrives at this position, every sensor can directly connect with the UAV to upload data. The CH is a helping node that aids in communication between UAV and sensors.

**Initialization**—The initialization algorithm is listed in Algorithm 2; it starts with sensors broadcasting a *hello* beacon with its global unique ID (GUID) and geo-location. The receiving sensor nodes form a list of neighboring nodes $L_{ngr}^{S}$ based on RSSI values. A sensor node populates a list $G_{ngr}^{S}$ of its all neighbors along with their geo-locations. Further, the node broadcasts its cost value to all neighbors. The cost of a sensor is calculated according to the number of neighbor sensors. According to HEED [56], the cumulative distance to all adjacent nodes is used to find the cost. A polygon-like structure of the sensor nodes is formed using Algorithm 3. The nodes calculate their central bias using this algorithm and measure the centroid via Paul Brourke’s equation [57]. In polygon formation Algorithm 3, the location information of all the neighbor sensors is used. The nodes having an RSSI value of more than a threshold can only participate in the polygon formation. This algorithm is developed by extending the monotone chain algorithm by Mei et al. [58]. The nodes are sorted in the start according to their geo-locations with respect to the x-axis [59]. Then two lists, $H_{L}$ and $h_{U}$, are formed, having the lower and upper hulls. The $H_{ngr}^{S}$ is formed by subtracting the $H_{L}$ and $H_{U}$ into two subsets, α and β, respectively, such that they have more than one element. The iteration is performed on all the nodes in the lists, and the nodes with the same directions are added. Finally, both lists of lower and upper haul are concatenated. Once a polygon-like structure is formed, the area is computed using Equation (7). According to the computed value probability Equation (11), the node is proclaimed as final $Ch_{p}$, or candidate $Ch_{\psi }$.

**Recurrence**—After the initialization phase, the algorithm starts the second phase called recurrence, as listed in Algorithm 2 (lines 10–29). In this phase, the cost of each CH in list $L_{CH}$ is compared, and the $CH_{\rho }$ with minimum cost is selected. If this is the current sensor node, broadcast a CH message with ID, cost, and update $CH_{p}^{temp}$ to TRUE. Otherwise, broadcast CH message keeping $CH_{p}^{temp}$ as FALSE. In the case where the CH probability is 1, broadcast $CH_{p}$ message by setting $CH_{p}^{temp}$ to TRUE, and if the probability is any number between 0 and 1, broadcast $CH_{\psi }$ message keeping $CH_{p}^{temp}$ as FALSE and repeat this process until $CH_{p}$ is equal to 1.

**Finalization**—In this stage, the sensors check its $CH_{p}^{temp}$ flag. In case it is *FALSE*, it acquires the $CH_{\rho }$ from $L_{CH}$ and sends a join request to that sensor node. In case the $CH_{p}^{temp}$ variable is *TRUE*, the sensor sends a hello message to all nearby nodes with CH status as $CH_{\psi }$. When a node receives a cluster join request, the node with status $CH_{p}$ adds the sensor to its CM list. However, if a node is $CH_{p}$, it broadcasts a message to let its neighbors discover the node.
**Algorithm 2** Clustering Algorithm**Input** $L_{ngr}^{S}\leftarrow \emptyset$                                                                                                ▹ Empty list of sensor’s neighbors**Output** $CH_{p}$                                                                                                            ▹ Finally selected cluster head1:$L_{ngr}^{S}\leftarrow GetList\left(neighbors\right)$                                                                               ▹ Obtain neighbors via RSSI2:$G_{ngr}^{S}\leftarrow GetGeoLocations\left(L_{ngr}^{S}\right)$                                                                      ▹ Obtain (x,y,z) coordinates3:$BroadcastCost(L_{ngr}^{S})$                                                                                             ▹ Send cost of all members4:$CreatePolygon(G_{ngr}^{S})$                                                                                                       ▹ Using Algorithm 35:Area←CalArea()                                                                                                            ▹ Using Equation (7)6:$\Theta_{x,y}\leftarrow CalCentroid\left(\right)$                                                                                                   ▹ Using Equation (10)7:$d\leftarrow CalDistance(this.GeoLoc,\Theta_{x,y})$                                                                   ▹ Calculate the distance8:$P_{CH}\leftarrow CalProbablity\left(\right)$                                                                                                ▹ Using Equation (11)9:$CH_{p}^{temp}\leftarrow FASLE$10:**do**11:    **if** $(L_{CH}\leftarrow \{$*c:* c is a CH }≠∅) **then**12:        $CH_{\rho }\leftarrow min\left(CalCost\left(L_{CH}\right)\right)$                                                   ▹ Choose CH from list with min cost13:        **if** $CH_{\rho }^{ID}=THIS.ID$ **then**14:           **if** $P_{CH}=1$ **then**15:               $Broadcast(THIS.ID,CH_{p},cost)$16:               $CH_{p}^{temp}\leftarrow TRUE$17:           **else**18:               $Broadcast(THIS.ID,CH_{\psi },cost)$19:           **end if**20:        **end if**21:    **else if** $P_{CH}=1$ **then**22:        $Broadcast(THIS.ID,CH_{p},cost)$23:        $CH_{p}^{temp}\leftarrow TRUE$24:    **else if** $Random\left(0,1\right)\leq P_{CH}$ **then**25:        $Broadcast(THIS.ID,CH_{\psi },cost)$26:    **end if**27:    $CH_{previous}\leftarrow P_{CH}$28:    $P_{CH}\leftarrow min\left(P_{CH}x2,1\right)$29:**while** $CH_{previous}=1$30:**if** $CH_{p}^{temp}=FALSE$ **then**31:    **if** $(L_{CH}\leftarrow \{$*c:* c is a $CH_{p}$}≠∅) **then**32:        $CH_{\rho }\leftarrow min\left(CalCost\left(L_{CH}\right)\right)$33:        $JoinCluster(CH_{\rho },THIS.ID)$34:    **else**35:        $Broadcast(THIS.ID,CH_{p},cost)$36:    **end if**37:**else**38:    $Broadcast(THIS.ID,CH_{p},cost)$39:**end if**

**Algorithm 3** Polygon Formation
**Input** $G_{ngr}^{S}$                                                                      ▹ List of nodes with locations, sorted w.r.t. x-axis
**Output** $H_{ngr}^{S}$                                                                     ▹ List of nodes participating in making polygon
1:$H_{U}\leftarrow \emptyset$ & $H_{L}\leftarrow \emptyset$                                                                                  ▹ List of upper and lower hulls2:**for** j=1 to $SIZE(G_{ngr}^{S})$ **do**3:    **while** ($\alpha \subset H_{L}$ & SIZE(α)>1) **do**4:        $DELETE(H_{L}\left[SIZE-1\right])$                                                                        ▹ Remove last element5:        $APPEND(H_{L}\left[G_{ngr}^{S}\left[j\right]\right])$6:    **end while**7:    **while** ($\beta \subset H_{U}$ & SIZE(β)>1) **do**8:        $DELETE(H_{U}\left[SIZE-1\right])$                                                                       ▹ Remove last element9:        $APPEND(H_{U}\left[G_{ngr}^{S}\left[j\right]\right])$10:    **end while**11:
**end for**
12:

$DELETE(H_{L}\left[SIZE-1\right])$

13:

$DELETE(H_{U}\left[SIZE-1\right])$

14:

$Return(H_{ngr}^{S}\leftarrow CONCAT\left(H_{L},H_{U}\right))$




**Algorithm 4** Cluster Head Position Discovery
**Input** $G_{CH}\leftarrow \emptyset$                                                                                                             ▹ Empty list of CHs with positions
**Output** $G_{CH}$                                                                                                                               ▹ List of CHs with positions
1:
**do**
2:    BroadcaseSearchCH()                                                                                                 ▹ Broadcast a CH search message3:    SetTimer()                                                                                                                      ▹ Set timer for next broadcast4:    Msg←Receive(Msg)                                                                                                 ▹ Receive informing message5:    **if** $Msg.Type=(CH_{information})$ **then**6:        $CH_{x-axis}\leftarrow Msg.X$7:        $CH_{y-axis}\leftarrow Msg.Y$8:        $CH_{z-axis}\leftarrow Msg.Z$9:        $CH_{position}\leftarrow \left\{CH_{x-axis},CH_{y-axis},CH_{z-axis}\right\}$                                                       ▹ Collect position values10:    **end if**11:    $G_{CH}.PUSH\left(CH_{position}\right)$                                                      ▹ Push received CH position information into list12:**while** TRUE


### 4.2. Data Scheduling

There is a *B* number of base stations (BS) located across the area. These BS are connected with *f* number of fog nodes. The UAV visits the CH’s to collect data and move towards the next CH. During that traveling, if the UAV reaches the range of a BS, it offloads the collected data and flushes its memory for later reuse. Once a BS receives data, the data are stored as a list of tasks in the local buffer. The BS further schedule the data processing to its connected heterogeneous fog nodes. This forwarding is based on the power efficiency and response time of the fog nodes.

**Scheduling Algorithm**—It initiated once the UAV completed the offloading process. The BS has a list of workloads and the connected fog nodes. Here, we extended Dispersive Stable Task Scheduling (DATS) [60]. As illustrated in Algorithm 1, for each workload ω in Ω, the BS calculates the availability index [45], response rate by Equation (22), and efficiency by Equation (21) of all fog nodes. We call these values initial demands, and the algorithm further optimizes these values for each workload. Each fog node, *f* in ϝ, the availability, response rate, and efficiency values are compared with the initial demands, and eligible nodes are inserted into $S_{A}$, $S_{R}$, and $S_{E}$. Once all fog nodes have been traversed, the intersection list *S* is calculated. The set *S* represents the list of all fog nodes that meet initial demands of availability, response rate, and efficiency. Further, the best node from the list is selected using Equation (23).

(22) $$Min\sum_{J=0}^{n-1}\left(S_{A}^{j},S_{E}^{j},S_{R}^{j}\right)$$

However, in case the set has no item, the value of the demand of efficiency is reduced by a factor κ and all fog nodes are re-traversed. This trade-off efficiency with availability and response rate helps in finding the optimal solution from available fog resources. Thus, only the efficiency is compromised in a case when no fog node is available that meets initial demand because we are giving more preferences to availability and response rate over energy consumption.

## 5. Evaluation

A smart farming case scenario is simulated using omnet++ (www.omnetpp.org, accessed on 14 June 2021) as a simulation tool. The sensors are deployed randomly across the farm; there exist base stations at the boundary of the farm, and dedicated fog nodes are connected with each BS. The list of simulation parameters and system parameters are given in Table 2 and Table 3, respectively. The size of the data packet is given in the table for processing, and the data packet is translated into million instructions per second (MIPS) for processing.

We compared the performance of the proposed clustering and data collection with HEED [56]. HEED, also known as Hybrid Energy-Efficient Distributed, is a clustering framework for sensors. HEED periodically selects the cluster head according to the residual energy of the sensors, whereas our technique selects the cluster head once and uses that information throughout all the rounds because we assume that sensors are equipped with renewable energy sources. We applied the HEED technique with UAV for data collection and compared results with the proposed technique in terms of the number of control packets, network delay, and energy consumption.

For workload processing, and scheduling at the fog servers, we compared the proposed system with a maximal energy-efficient task scheduling (MEETS) algorithm [40]. In MEETS, the energy efficiency is improved for homogeneous fog networks and nearby available fog resources. However, we believe that along with energy node availability, network response time also plays an important role for a latency-sensitive fog network environment.

**Figure 2:**
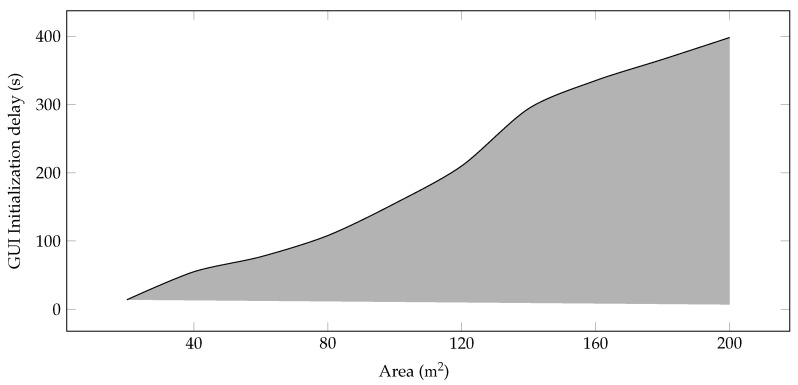
Component initialization delay in GUI mode.

**Components Initialization Delay in GUI Mode**—The proposed model is evaluated in OMNeT++ in GUI mode. However, a significant component and interface initialization delay is observed, and this delay increases with the increase in farm area as more sensors and devices are installed. Figure 2 shows that this delay varies from 14 to 390 ms for the 200 m${}^{2}$ area.

**Figure 3:**
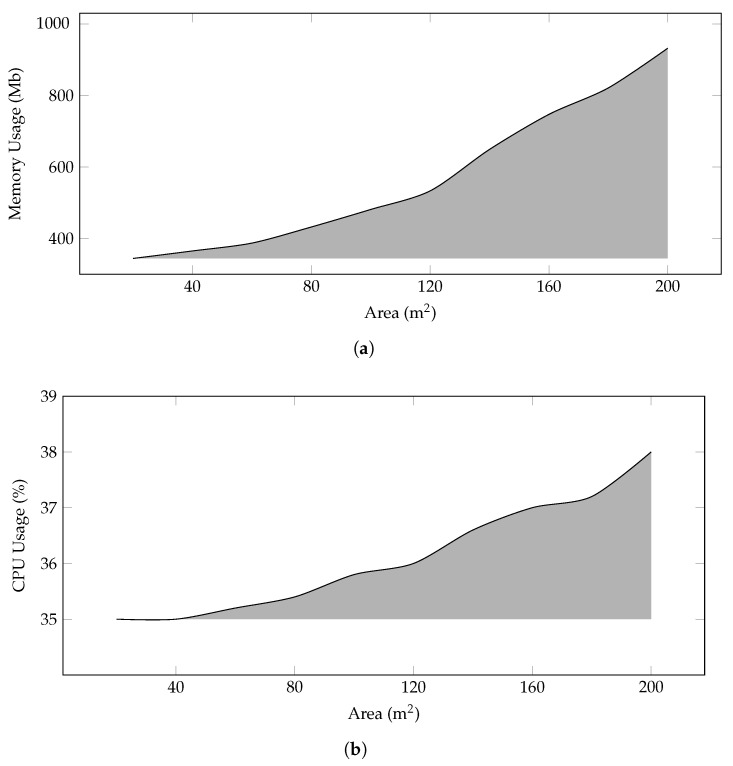
Average memory and CPU usage of system according to number of nodes and request rates (μ). (**a**) Memory usage (**b**) CPU usage.

**CPU and Memory Usage**—Figure 3 shows the CPU and Memory analysis of the system with varying farm sizes. As per the proposed model, the increase in the area of farms brings about a sufficient increase in the number of deployed sensors, BS, and fog servers. During the simulation, each node reserves some space in the memory, and this space increases with the increase in the area as shown in Figure 3a. The framework creates and disseminates a lot of messages that include beacons, control, and data messages. This process affects the processing, and the CPU usage is given in Figure 3b.

**Figure 4:**
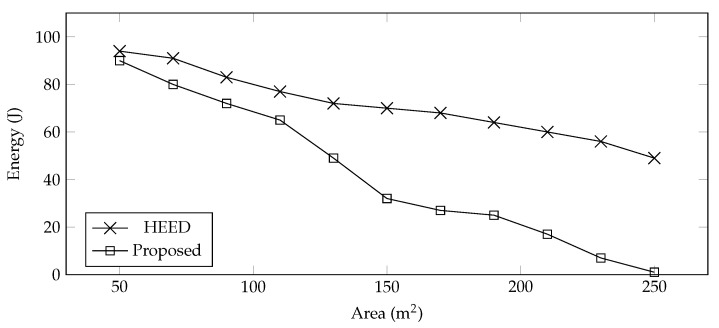
Energy consumption vs. farming area.

**Energy Consumption**—The energy consumption of the proposed system is compared with HEED by varying the farming sizes. The traditional HEED algorithm consumes more energy as compared to the proposed system, as shown in Figure 4. In HEED, the UAV explores to find the CHs, whereas in the proposed system, the positions of the CH are collected first, and then an optimized trajectory is designed to efficiently utilize the energy. Furthermore, the UAVs follow the calculated trajectory, collect data, and update the system about dead nodes. Furthermore, the farming area used for the evaluation is of square shape, and sensors are deployed randomly. Now, with the increase in area, the inter-cluster distance is also increased, and the energy consumption is measured in terms of cost of data transmission which increases with the intra-cluster distance.

**Figure 5:**
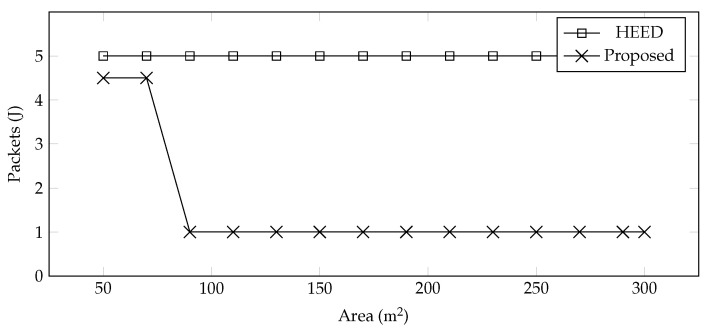
No. of Rounds vs. Control Packets.

**Control Packets**—The number of control packets shared during clustering is very important because energy can be preserved through reduced communication. In the proposed scheme, the clustering is performed only once, and this information is used for data collection in all rounds. We compared these control messages with HEED as shown in Figure 5, and it is observed that HEED sends more control packets as compared to the proposed system.

**Figure 6:**
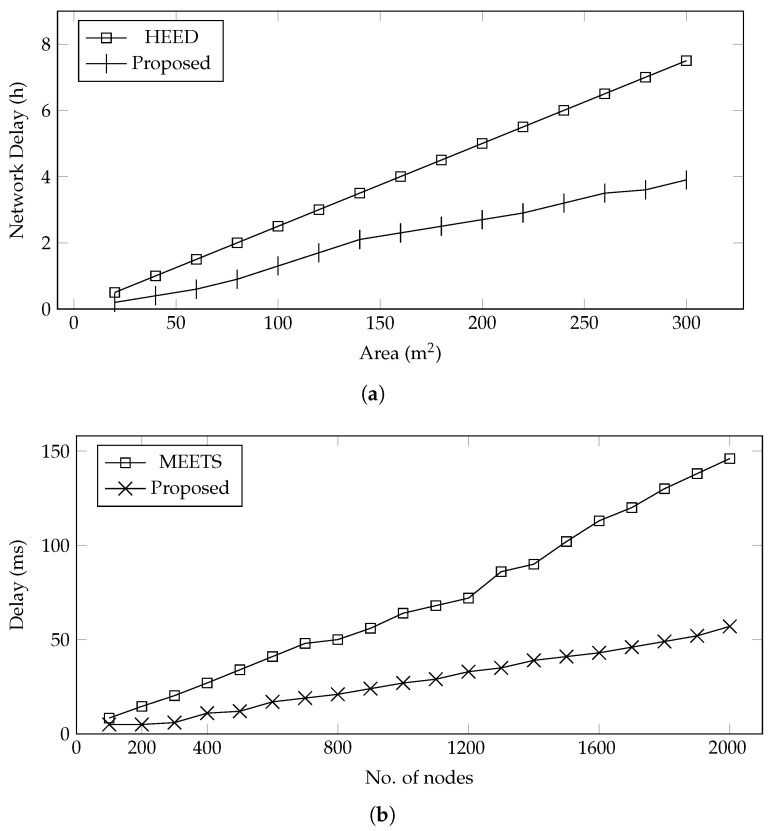
Data collection delay with varying farming area, and data processing delay with varying sensors. (**a**) Data collection delay vs. area; (**b**) data processing delay vs. no. of nodes.

**Network Delay**—The network delay is separated for data collection and data processing, as shown in Figure 6. The delay in data collection is compared with HEED, and it is observed that the proposed system outperformed in terms of network delay, as shown in Figure 6a. The delay from data processing is compared with MEETS, and it is also observed that the proposed scheduling algorithm outperformed, as shown in Figure 6b. The primary reason for this performance is that the proposed framework selects the CH only once when needed, whereas HEED gradually runs the CH selection algorithm that causes network congestion and hence increases delay. Another reason is, in HEED, data are collected via relaying through CH, whereas the proposed technique collected data directly from the sensors.

## 6. Conclusions

In this article, we have proposed an energy-efficient, UAV-assisted data collection technique from randomly deployed sensors in the farming area. We first clustered the randomly deployed sensors based on their RSSI values and selected cluster heads. Then, using Dijkstra and GA, an optimized trajectory is obtained, and the trajectory data are thus collected from the sensors. We further proposed a multi-objective fog-based data processing framework for data scheduling and processing at multiple fog servers. The performance of the simulation is evaluated by simulation in omnet++, and results are compared with HEED and MEETS for data collection and data processing, respectively. It is observed that the proposed framework outperformed in terms of network delay, energy efficiency, CPU-Memory usage, and the number of control packets.

## Figures and Tables

**Table 1:** Summary of notations.

Sr.	Symbol	Definition
1	*S*	List of sensor nodes of size *N*
2	*X*	List of positions in $R^{3\times 1}$ space
3	$U_{start}$	Starting position of the farm
4	$U_{final}$	Final/Ending position of the farm
5	$\Upsilon_{U}$	The initial height of the UAV
6	$\varrho_{Sr}$	The transmission radius of sensors
7	$\varrho_{Ur}$	The transmission radius of UAV
8	$br_{p}$	The time of previous hello message
9	$br_{n}$	The time of next hello message
10	$\upsilon_{U}$	The speed of UAV
11	$U_{R}^{Q_{y}/2}$	The range of transmission
12	$\left(\frac{U_{R}^{Q_{y}/2}}{\upsilon_{U}}\right)$	The duration when UAV and sensors are in range
13	$\Theta_{x,y}$	The centroid of the polygon/cluster
14	$G^{X}$	List of nodes’ x-axes located at the edge
15	$G^{Y}$	List of nodes’ y-axes located at the edge
16	*K*	No. of nodes contributing to polygon formation
17	Area	Area of the polygon
18	*B*	No. of base stations
19	*f*	No. of nodes connected with BS
20	$P_{CH}$	Probability of a sensor to become CH
21	ζ	Normalization factor for iterations
22	$\chi_{c,n}$	Distance between current and central node
23	$CH_{p}$	Final selected cluster head
24	$CH_{\psi }$	Node eligible for cluster head
25	$CH_{\rho }$	The optimal CH before selecting the final CH
26	$L_{ngr}^{S}$	List of sensor’s neighbors
27	$H_{ngr}^{S}$	List of nodes participating in making polygon

**Table 2:** Simulation parameters.

#	Parameter	Value/Description
1	Farming area	250 × 250 m
2	Sensors	2000
3	Base stations	5
4	Fog servers in each BS	5–10
5	Packet size (data)	8 KB
6	Packet size (hello)	100 B
7	Initial altitude (UAV)	40 m
8	Initial altitude (sensors)	0–4 m
9	Mobility (UAV)	Dynamic
10	Mobity (sensors)	Static
11	Speed (UAV)	25 m/s
12	Fog node capacity (computation)	1000 MIPS
13	MAC protocol	TDMA, CSMA

**Table 3:** System parameters.

#	Component	Version/Value
**Computer (CPU and Memory)**		
1	Processor	Intel(R) $Core^{TM}$ i5 1 GHz
2	Core(s)	4
3	Threads	8
4	Memory	16 GB
5	Operating system	Ubuntu 16.04 LTS
**Tools and Technologies**		
6	Omnet++	4.6
7	INET	3.2.4
